# The benefits of nurturant-involved parenting for children’s internalizing symptoms and cardiometabolic health in high-risk contexts

**DOI:** 10.1017/S0954579423000652

**Published:** 2023-06-30

**Authors:** Elizabeth R. Wiggins, Julie M. Brisson, Justin A. Lavner, Katherine B. Ehrlich

**Affiliations:** 1 Department of Psychology, University of Georgia, Athens, GA, USA; 2 Center for Family Research, University of Georgia, Athens, GA, USA

**Keywords:** cardiometabolic health, discrimination, internalizing symptoms, parenting, stress

## Abstract

Despite evidence that nurturant-involved parenting is linked with children’s social, psychological, and physiological development, less is known about the specific contexts in which nurturant-involved parenting is most beneficial for children’s mental and physical health. The present study examined how associations between nurturant-involved parenting and children’s internalizing symptoms and cardiometabolic risk varied as a function of children’s stress and discrimination. Participants included 165 Black and Latinx children (*M*_age_ = 11.5 years) and their guardians. Children reported on their ongoing stress, experiences of discrimination, and internalizing symptoms (depression and anxiety). Guardians provided information about their nurturant-involved parenting practices. Children’s cardiometabolic risk was assessed as a composite reflecting a high level of systolic or diastolic blood pressure, waist circumference, HbA1c, triglycerides, and low HDL cholesterol. Regression analyses indicated that among youth who reported high levels of stress and discrimination, nurturant-involved parenting was negatively associated with cardiometabolic risk. Although children’s stress and discrimination were significantly associated with their internalizing symptoms, neither stress nor discrimination moderated the relation between nurturant-involved parenting and internalizing symptoms. Results highlight the significant role that parents play in shaping children’s health, particularly among youth experiencing high levels of stress and discrimination.

The quality of parenting that children receive profoundly affects children’s lives and can have both short-term and long-term effects across social, psychological, and biological domains. Parenting behaviors such as nurturant-involved parenting, which is defined as high parental warmth, inductive reasoning, communication, and child monitoring (Brody et al., 2001), can be particularly beneficial for children’s well-being. Parental warmth has been positively associated with children’s psychological adjustment in culturally diverse samples (Khaleque, 2013) and negatively associated with children’s internalizing symptoms among a sample of primarily Hispanic youth (del Barrio et al., 2016). Similarly, supportive parenting has been shown to be positively associated with children’s cardiovascular functioning and negatively associated with inflammation in childhood (Bell & Belsky, 2008; Carroll et al., 2013; Miller et al., 2014). Despite evidence of the positive effects of nurturant-involved parenting on children’s development, some researchers have speculated that there are limits to the extent that parenting can influence children’s physical and psychological well-being (Ehrlich & Cassidy, 2021; Ehrlich, 2019). Accordingly, questions remain about the specific contexts in which nurturant-involved parenting is most beneficial for children’s mental and physical health.

Some research suggests that the benefits of nurturing and involved parenting practices are not uniform across contexts (Brody et al., 2001; Soenens et al., 2015). These benefits may differ in part because children’s development is influenced by complex interactions between children and the people and environments around them. Specifically, these interactions vary based on the joint characteristics of the child and the more immediate (e.g., home life) and distant (e.g., community norms) environments in which the interactions take place, including the changes that occur to that environment over time (Bronfenbrenner & Morris, 1998). In other words, the nurturant-involved parenting behaviors that primary caregivers employ may have different effects on children’s development and well-being depending on the contexts in which they occur.

For example, Ogg and Anthony (2020) found that only children from low-socioeconomic status (SES) backgrounds benefitted academically from parental warmth. For children living in more well-resourced environments, parental warmth was negatively related to their academic growth. Similarly, in a primarily White sample, parental monitoring was associated with fewer externalizing and antisocial behaviors among children who lived in unsafe neighborhoods (Pettit et al., 1999). Similar benefits of parental monitoring emerged in another study of Black and White children who spent a significant amount of time unsupervised (Laird et al., 2010). Conversely, parental monitoring and vigilance were unrelated to children’s health in a racially and socioeconomically representative sample of the United States (Corallo et al., 2023) and have been shown to be detrimental in lower-risk settings among both Black and White youth (Gonzales et al., 1996; Laird et al., 2010). The benefits of involved parenting on adolescent problem behaviors (delinquency, school-related problem behavior, and depressive symptoms) may be most evident among children living in unsafe neighborhoods, as Roche et al. (2007) found in a sample of Black and Latinx youth. When youth experience significant stress, there may be more opportunities for nurturing and involved parents to make overt demonstrations of care for their children, which can result in significant benefits for children’s well-being. These findings suggest that parenting may be most influential in high-risk settings (i.e., when children are exposed to significant stressors), and parenting may have less detectable benefits in low-risk contexts.

Several researchers have expanded on Bronfenbrenner’s theory of ecological development to more fully account for the influence of culture and racial identity (Garcia-Coll et al., 1996; Santos & Toomey, 2018; Velez-Agosto et al., 2017). These updated models suggest that Bronfenbrenner’s theory alone does not adequately capture the effects of racism, discrimination, and prejudice on children’s development. Racism and structural oppression can influence children’s lived experiences from the microsystem to the macrosystem, and the stressors that children face are affected by their racial identity. Garcia-Coll and colleagues (1996) propose that theoretical frameworks of development must incorporate constructs that are specific to populations of color as well as more general constructs that might vary depending on individual factors. Motivated by these frameworks, we explore how connections between parenting and health are affected both by children’s general stress and by their experiences of discrimination.

## Stress in childhood and adolescence

Childhood and adolescence are periods of development characterized by socioemotional change as youth construct their sense of identity, learn how they fit in with those around them, and adjust to school environments. Prior research has identified early adolescence as a period during which children are particularly vulnerable to negative health consequences associated with stressful life events (Romeo, 2013; Siegel & Brown, 1988). It is also during these stages of development that many mental and physical health disorders begin to emerge (Dietz, 1997; Kessler et al., 2007) making it an ideal time to examine associations between parenting and children’s outcomes, and the contexts in which these associations are strongest.

During childhood and adolescence, youth face many stressors, including peer pressure, family conflict, and increased pressure to succeed in school. Children and adolescents consistently report school as a major source of stress in their lives (Östberg et al., 2015; Anniko et al., 2019). Additionally, social stress from peers and parents can be highly stressful for children. Peer pressure is associated with a range of adverse health behaviors, including risk taking and substance use (Allen et al., 2006), and susceptibility to peer pressure has been linked with depressive symptoms (Santor et al., 2000). The family environment also continues to play a central role in children’s social worlds, and family conflict during childhood and adolescence is similarly associated with internalizing symptoms and health-related outcomes (Ehrlich et al., 2015a, 2015b; Formoso et al., 2000; Reinherz et al., 2003; Weymouth et al., 2016). Together, these sources of stress are salient features of children’s social worlds and may moderate the associations between nurturant-involved parenting and children’s mental and physical well-being.

In addition to facing these general stressors, children of color disproportionately experience unique psychological stress exposures in the form of discrimination. These exposures accumulate over time and increase risk of poor mental and physical health (Clark et al., 1999; Sims et al., 2012). Experiences of racial discrimination are alarmingly frequent in children’s lives, have increased in recent years, and are distressing interactions that children can recognize from a very early age (Dulin-Keita et al., 2011; Elenwo et al., 2022). Recent estimates suggest that by the time Black children reach adolescence, they report an average of five encounters of racial discrimination per day (English et al., 2020). The ubiquity of these experiences in childhood and adolescence highlights the importance of research considering the effects of discriminatory stress.

The experience of racial and ethnic discrimination among Black and Latinx children predicts poorer psychological well-being, including increased risk of depression and anxiety (Bernard et al., 2022; Clark et al., 2004; Gaylord-Harden & Cunningham, 2009; Lavner et al., 2023; Umaña-Taylor & Updegraff, 2007). In acting as a social stressor, exposure to discrimination in childhood and adolescence may also affect youths’ physical health by altering the functioning of biological stress-regulatory systems (Geronimus et al., 2006). Although experiences of racial discrimination have been associated with a range of cardiometabolic risk markers among adults, few studies have examined these associations in childhood. Evidence from adult samples suggests that experiences of racial discrimination are linked with coronary artery calcification and inflammatory markers such as high C-reactive protein levels and high interleukin-6 levels (Kershaw et al., 2016; Lewis et al., 2010). Additional research is needed to determine how experiences of discrimination in childhood may influence the association between nurturant-involved parenting and the physical health of children of color.

## The present study

Although prior research has led to significant advances in our understanding of how parenting practices affect children’s mental and physical health, several gaps remain. Most research on stress, discrimination, and health focuses on adult populations. Few studies have examined associations among parenting, stress, discrimination, and health in young children, and even fewer studies have examined these associations specifically in young children of color. In the present study, we address these limitations by examining these links in a sample of Black and Latinx children ranging in age from 8 to 16. This sample represents an understudied and historically marginalized population of youth who experience higher levels of stress in their daily lives, relative to lower-risk community samples. Additionally, most prior work has studied mental health and physical health outcomes in different study samples, leaving questions about whether the benefits of nurturant-involved parenting would apply within the same sample of children when exposed to the same environmental conditions. The present study addresses calls to examine mental and physical health outcomes within the same study (Ehrlich et al., 2016), providing a more comprehensive picture of the role of parenting in shaping children’s mental and physical health.

The primary purpose of this investigation is to examine the specific contexts in which supportive parenting is most beneficial for children’s health. Based on this broader goal, this study addressed the following research question: Are the benefits of nurturant-involved parenting most likely to emerge in high-risk contexts, when children are experiencing high levels of stress or discrimination? We hypothesized that among children experiencing high levels of stress or discrimination, nurturant-involved parenting would be negatively associated with internalizing symptoms and cardiometabolic risk. In contrast, we hypothesized that the associations between nurturant-involved parenting and children’s health would be attenuated or even non-significant among children experiencing low levels of stress or discrimination.

## Method

### Participants

Participants included 165 youth (*M*_age_ = 11.5 years, *SD* = 2.6 years; 49.1% girls; 60% Black/African American; 40% Hispanic/Latinx) and their guardians from the Athens, GA area. Families were recruited using flyers, sharing information at community events, working directly with community liaisons, and using snowball recruiting through participants already enrolled in the study. To be eligible, children had to identify as Black/African American or Hispanic/Latinx, be between the ages of 8–16 years, and be fluent in English. Primary caregivers who were not fluent in English were able to participate in the study with assistance from Spanish-speaking research assistants. Youth were excluded if they had a history of any chronic diseases (e.g., diabetes, an autoimmune condition), were receiving dialysis or other blood-related treatment, or had been diagnosed with any severe cognitive disabilities or developmental delays. If children had recently experienced an acute infection, their visit was scheduled at least four weeks after the infection. Two children were ultimately excluded due to one child having uncontrolled diabetes (hemoglobin A1C [HbA1c] value of 15.7%), and one family being unable to complete the study procedures, resulting in a final analytic sample of 165. Over half of the sample (57%) met the 2021 poverty threshold (HHS, 2021), and 26% of guardians in the sample did not complete high school.

The Institutional Review Board at the University of Georgia approved all study protocols. Guardians (93% mothers, 4% fathers, and 3% other) provided written consent, and youth provided written assent. Children and their guardian each received $50 for participating in the study visit along with $20 for transportation.

### Procedure

All data were collected between July 2021 and May 2022. Families in the study completed baseline assessments as part of a larger study focused on risk and resilience among at-risk youth. During the study visit, trained research assistants administered surveys to children and guardians. To aid in comprehension, research assistants read survey questions out loud to younger children who requested assistance. For Spanish-speaking parents, measures were translated, and a Spanish-speaking research assistant was available to clarify any questions. Research assistants completed biometric assessments of children’s height, weight, waist and hip circumference, and blood pressure. Participants also completed assessments beyond the scope of the current study. Certified pediatric phlebotomists performed all blood draws.

### Measures

#### Stress

Youth reported on their perceived stress using subscales from the Adolescent Stress Questionnaire (Byrne et al., 2007) assessing the stress of home life, stress of peer pressure, and stress of performing well in school. We included a subset of 11 questions from these scales that were age-appropriate and relevant to the present study. Questions reflected common stressors in children’s lives, including “arguments at home,” “keeping up with schoolwork,” and “being judged by your friends.” Responses were rated on a Likert-type scale from 1 (*not at all stressful or is irrelevant to me*) to 5 (*very stressful*). Items from subscales were combined to generate an overall mean stress score (α = .84, *M*[*SD*] = 2.38 [.84]), with higher scores reflecting greater stress.

#### Discrimination

Youth reported on discrimination using the Everyday Discrimination Scale (Williams et al., 1997), which is a 9-item measure designed to assess routine experiences of unfair treatment. The scale is validated for use with children as young as 7 (e.g., Dulin-Keita et al., 2011) and includes items such as “you are treated with less respect than other people are” and “people act as if they’re better than you are.” Responses were scored on a 6-point Likert-type scale ranging from 1 (*almost every day*) to6 (*never*). Scores were subsequently reversed so that higher scores represented greater discrimination. Responses were averaged to generate an overall mean discrimination score (α = .82, *M*[*SD*] = 2.28 [1.08]).

#### Nurturant-involved parenting

Guardians completed the Nurturant-Involved Parenting Scale (Brody et al., 2001) to assess parental warmth, involvement, low hostility, and low rejection. This 9-item scale includes responses ranging from 1 (*never*) to 4 (*always*). Sample items include “how often does your child talk to you about things that bother him/her?” and “how often do you ask your child what he/she thinks before deciding on family matters that involve him/her?” Responses were averaged to generate an overall mean score (α = .76, *M*[*SD*] = 3.33 [.45]), with higher scores reflecting more nurturant-involved parenting.

#### Internalizing symptoms

To assess depressive symptoms, youth completed the Center for Epidemiologic Studies – Depression short form (CES-D; Andresen et al., 1994; Radloff, 1977). This 10-item questionnaire asks youth about their depressive symptoms over the course of the past week. Responses were scored on a 4-point Likert-type scale, ranging from 0 (*rarely or none of the time; less than one day*) to 3 (*all of the time; 5–7 days*), and averaged to create a mean score (α = .69, *M*[*SD*] = .80 [.48]), with higher scores reflecting more depressive symptoms. Youth reported on their anxiety using the generalized anxiety subscale of the Spence Children’s Anxiety scale (SCAS; Spence, 1997). This 6-item subscale is validated for use with children and adolescents and includes items such as “when I have a problem, I get a funny feeling in my stomach” and “I worry that something bad will happen to me.” Response options ranged from 0 (*never*) to 3 (*always*), which were averaged to create a mean score (α = .80, *M*[*SD*] = 1.1 [.71]); higher scores reflected greater anxiety symptoms. The means of these measures in our sample are comparable to the means reported in other studies that have assessed mental health in youth (e.g., Buchan et al., 2021; Spence, 1997). Due to the high correlation between the two scales (*r* = .58, *p* < .001), we standardized the means from each scale and then averaged those scores to create a composite that reflects children’s internalizing symptoms.

#### Cardiometabolic risk

Cardiometabolic risk was measured using the following criteria: systolic and diastolic blood pressure (BP), waist circumference, high-density lipoprotein (HDL) cholesterol, triglycerides, and HbA1c. Research assistants collected three readings for children’s blood pressure (OMRON IntelliSense Blood Pressure Monitor HEM-907XL). Because the first reading can be elevated, only the final two readings were averaged to create BP estimates (Negroni-Balasquide et al., 2016). BP cutoffs were determined based on age, sex, and height percentiles, following guidelines from the American Academy of Pediatrics (AAP; Flynn et al., 2017). Children under 13 years old met the cutoff if their systolic or diastolic blood pressure was ≥90th percentile. Children ages 13 and older met the cutoff if their systolic BP was ≥120 or their diastolic BP was ≥80.

Researchers measured waist circumference twice at the midpoint of the upper iliac crest and lower costal margin at the midaxillary line. If the first two measurements were more than 0.2 cm apart, then researchers measured waist circumference a third time. The average of the closest two readings represented the mean. Waist circumference cutoffs were determined based on age and sex percentiles following guidelines from the International Diabetes Federation (Alberti et al., 2007; Fernández et al., 2004).

Children were asked not to eat or drink for two hours prior to the appointment. Following the blood draw, samples were couriered to the local hospital’s CLIA certified laboratory for analysis. HDL cholesterol and triglycerides were assessed using standard enzymatic techniques on a Beckman Coulter AU5800 chemistry analyzer. HbA1c was measured using the Bio-Rad D-10. We used established cutoffs (Alberti et al., 2007; Zimmet et al., 2007) to represent low HDL cholesterol (<40 mg/dL), elevated triglycerides (≥150 mg/dL), and elevated HbA1c (≥5.7%).

We created dichotomous variables for each component (systolic and diastolic BP, waist circumference, HDL cholesterol, triglycerides, and HbA1c) reflecting whether participants met the specified cutoffs. There was considerable variability in the number of children who met the threshold for each cardiometabolic risk component: blood pressure: 19%; waist circumference: 35%; HDL cholesterol: 12%; triglycerides: 15%; HbA1c: 21%. These percentages are comparable to other samples assessing cardiometabolic risk in youth (e.g., Andes et al., 2020; Christian et al., 2011; Harrington et al., 2013; Jackson et al., 2018; Nguyen et al., 2015). The dichotomized components were summed to generate an overall cardiometabolic risk sum score with a possible range from 0 to 5, where higher scores indicated greater risk. In this sample, scores ranged from 0 to 4 (*M* = 1.02, *SD* = .93); 54 youth had no components that reflected cardiometabolic risk, 68 youth had one component, 31 youth had two components, 10 youth had three components, and 2 youth had four cardiometabolic risk components.

#### Covariates

All models controlled for age, sex, race/ethnicity, and socioeconomic status (SES) risk. Age was represented by the child’s age (in years) at the time of the study visit. Sex was coded as 0 = *male* and 1 = *female*. Race was coded as 0 = *Black/African American* and 1 = *Hispanic/Latinx*. Drawing on previous research (Brody et al., 2018), we generated an SES risk composite based on six components: poverty based on federal guidelines, primary caregiver unemployment, receipt of government assistance (e.g., Temporary Assistance for Needy Families [TANF], Supplemental Nutrition Assistance Program [SNAP] benefits), primary caregiver single parenthood, primary caregiver education level less than high school graduation (or GED equivalent), and caregiver reported inadequacy of family income. We created dichotomous variables for each component to generate an SES risk index, with possible and observed scores ranging from 0 to 6 (*M* = 2.57, *SD* = 1.39).

### Data analysis

Analyses were conducted using SPSS Version 28.0.1.1. Moderation analyses were tested using Hayes’ PROCESS Macro (v4.1, Model 1). Each model included the main effect of nurturant-involved parenting, the main effect of a stressor (general stress or discrimination), the interaction between nurturant-involved parenting and the stressor, and the covariates. There were a total of four models, one for each stressor, run separately for each of the two outcomes (mental and physical health). Continuous variables were centered prior to analysis. We probed interaction effects using the Johnson–Neyman technique (Johnson & Fay, 1950) to determine specific regions of significance. Table 1 presents descriptive statistics and bivariate correlations for the sample.

## Results

### Nurturant-involved parenting, children’s stress, and children’s health

#### Internalizing symptoms

In this model, there was a significant positive association between children’s self-reported stress and their internalizing symptoms, with a medium effect size (see Table 2). Guardian reports of their nurturant-involved parenting were not significantly associated with children’s internalizing symptoms; similarly, and contrary to our hypothesis, the interaction between children’s stress and nurturant-involved parenting was not significant.

#### Cardiometabolic risk

**Figure 1.**
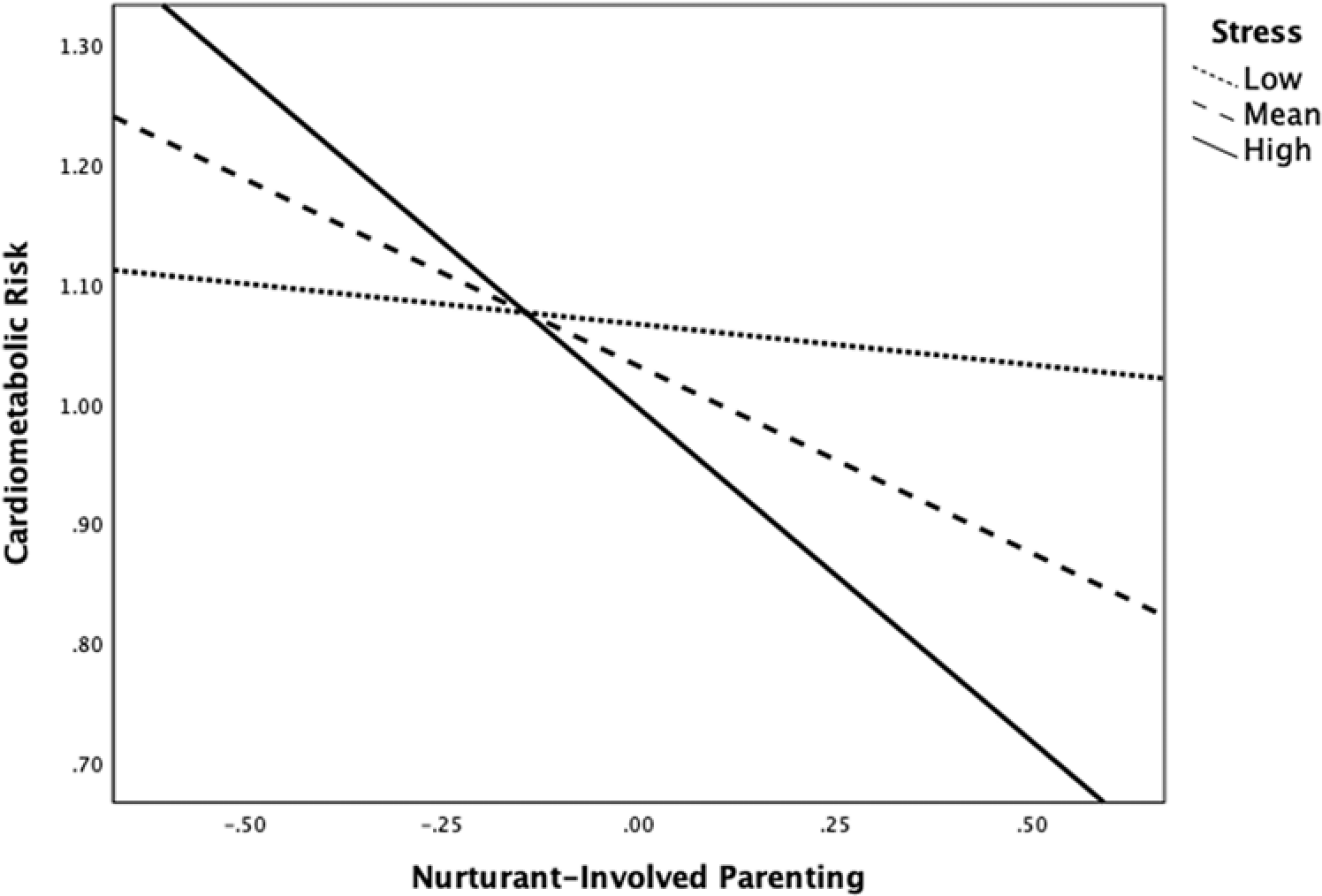
Interaction between nurturant-involved parenting and children’s stress on children’s cardiometabolic risk. *Note.* The figure shows estimated regression lines at *±*1SD and mean of children’s self-reported stress.

As shown in Table 2, in this model there was a marginally significant main effect of nurturant-involved parenting that was qualified by a marginally significant nurturant-involved parenting × stress interaction (*b* = −.37, *SE* = .20, *p* = .06). We probed this interaction and found that for children whose mean stress scores were more than .02 standard deviations above the mean (40.7% of the sample), nurturant-involved parenting was significantly negatively associated with cardiometabolic risk (see Figure 1). Nurturant-involved parenting was not significantly associated with cardiometabolic risk for youth with self-reported stress values below the .02SD threshold.

### Nurturant-involved parenting, children’s discrimination, and children’s health

#### Internalizing symptoms

In this model, there was a significant positive association between children’s self-reported experiences of discrimination and their internalizing symptoms, with a small effect size (see Table 3). As with the findings for children’s stress, guardian reports of their nurturant-involved parenting were not significantly associated with children’s internalizing symptoms and the interaction between children’s experiences of discrimination and nurturant-involved parenting was not significant.

#### Cardiometabolic risk

**Figure 2.**
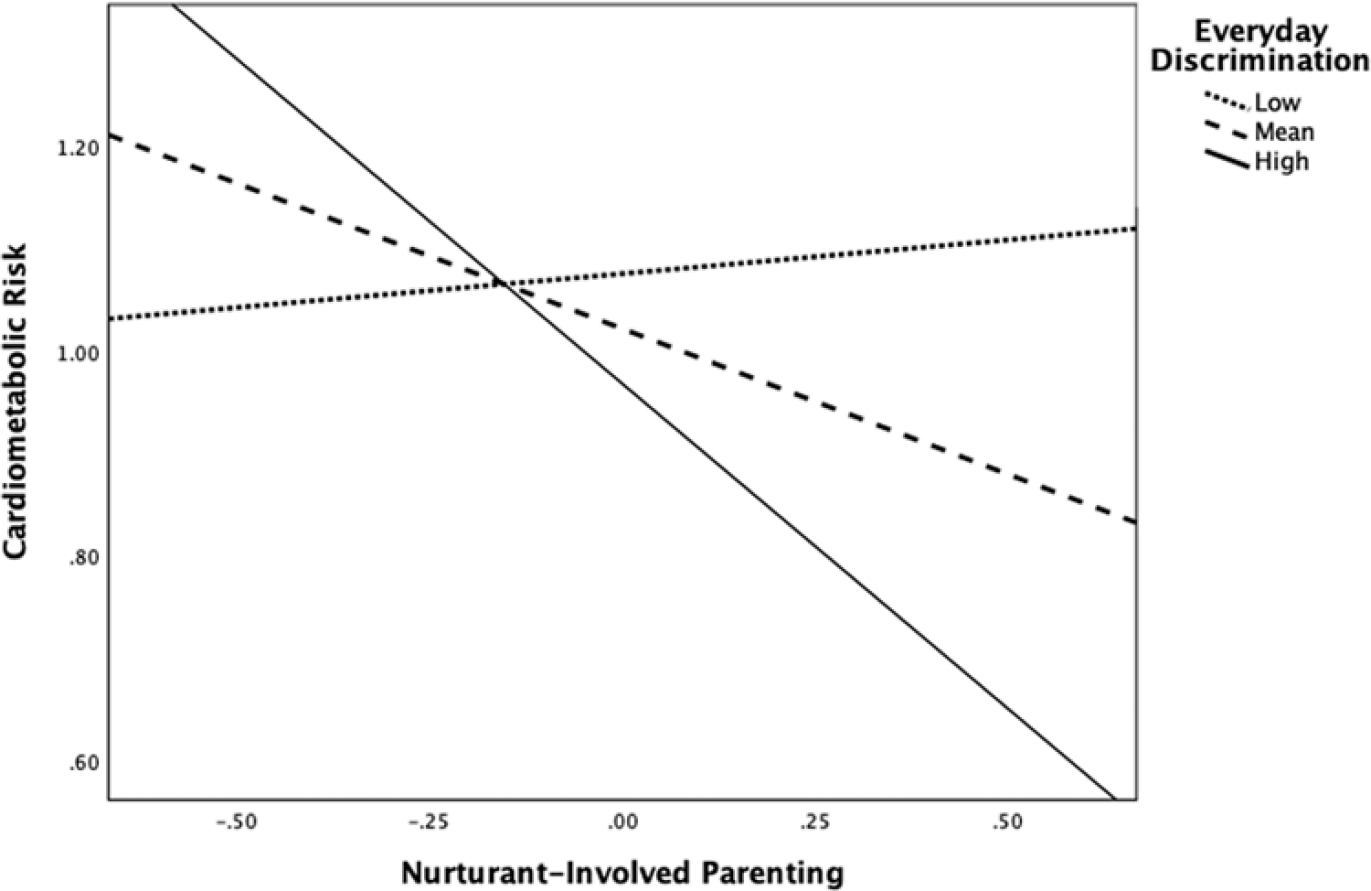
Interaction between nurturant-involved parenting and children’s experiences of discrimination on children’s cardiometabolic risk. Note. The figure shows estimated regression lines at ±1SD and mean of children’s self-reported everyday discrimination experiences.

Although there were no main effects of nurturant-involved parenting or discrimination on cardiometabolic risk in this model, there was a significant nurturant-involved parenting × discrimination interaction (*b* = −.32, *SE* = .16, *p* = .04). Probing of the interaction indicated that nurturant-involved parenting was negatively associated with cardiometabolic risk when average reports of discrimination were more than .11 standard deviations above the mean (38.0% of the sample; see Figure 2). Nurturant-involved parenting was not significantly associated with cardiometabolic risk below the .11SD threshold.

## Discussion

This study examined the effects of nurturant-involved parenting, stress, and discrimination on children’s mental and physical health within a sample of Black and Hispanic/Latinx 8–16-year-old children. Results revealed a positive association between children’s experiences of stress and discrimination and internalizing symptoms. Partially supporting our hypothesis, we found that nurturant-involved parenting was significantly negatively associated with children’s cardiometabolic risk when children reported higher levels of stress and discrimination and not when they reported lower levels of stress and discrimination. There was not evidence for this pattern for children’s internalizing symptoms, however.

The association between nurturant-involved parenting and cardiometabolic risk significantly varied as a function of stress and discrimination. Our findings are consistent with previous research suggesting that nurturant-involved parenting may matter most for children’s physical health in high-stress contexts (Laird et al., 2010; Ogg & Anthony, 2020; Pettit et al., 1999). Although supportive parenting is thought to be beneficial for all children, the health benefits of nurturant-involved parenting may be most evident when children are faced with significant stress. When children experience high levels of stress and/or discrimination, they may engage in conversations with parents that help relieve discomfort and promote healthy coping mechanisms, as opposed to engaging in health behaviors (e.g., substance use, caloric consumption) that might increase their cardiometabolic risk. Notably, similar patterns and effect sizes emerged across both general stressors and discriminatory stress, reinforcing the notion that considering the broader context is important when examining links between parenting and physical health.

Unexpectedly, our findings suggested that the benefits of nurturant-involved parenting in high-risk settings were limited to physical health, but not mental health. As previously noted, stress and discrimination are linked to poor health behaviors and coping mechanisms, such as substance use, sleep, activity, and caloric consumption (Ng & Jeffery, 2003; Pascoe & Richman, 2009; Schneiderman et al., 2005; Torres & Nowson, 2007). As a result, we may see the benefits of nurturant-involved parenting more clearly with cardiometabolic risk in comparison to internalizing symptoms. Future research examining more robust measures that tap into possible health behavior pathways could shed light on why parenting is promotive for physical health but not mental health when children face stress in their social worlds. Given the prevalence of these chronic stressors and discriminatory experiences in children’s lives, it will be important to identify what promotes youth mental health in these challenging environments.

There are several mechanisms that may explain why the benefits of nurturant-involved parenting on physical health and well-being may be strongest in high-risk contexts, and these factors should be examined in future research. First, when parents and children have a history of open communication and parents are able to provide effective support, children may experience more relief from stressful environments and engage in fewer negative coping strategies compared to children without a parent they can talk to about difficult experiences (Howard & Medway, 2004). As a result, they may be able to derive benefits from this support that translate into better physical health. Second, these discussions with parents might serve as opportunities for children to learn helpful strategies from their parents about how to navigate a stressful or hostile world. Children can then use these strategies throughout their lives as they encounter additional stressors. Within the context of a supportive parent–child relationship, children are likely to not only feel comforted in the moment, but to learn healthy and effective strategies from discussing challenging and unpleasant experiences with their caregivers that help them better cope with future stressors.

Although there were not significant effects of nurturant-involved parenting on children’s internalizing symptoms, children reporting higher levels of stress and higher levels of discrimination reported greater internalizing symptoms. This finding converges with other work showing that these experiences take a significant toll on children, even at an early age. As youth get older, they encounter more stressors at home, with peers, and at school. These stressors are linked to children’s mental health, as our study and others indicate (e.g., Allen et al., 2006; Reinherz et al., 2003). The stress of discrimination among the children in our sample was also significantly associated with their mental health, consistent with previous findings (Benner et al., 2018). Previous work related to the biopsychosocial model of racism (Clark et al., 1999) asserts that experiences of racial discrimination lead to psychological stress responses, including feelings of anger, resentment, helplessness, and hopelessness. These emotional responses to discrimination can subsequently result in increased internalizing symptoms, such as depression and anxiety. Our findings are in line with existing research linking stress and discrimination with poor mental health; however, it is important to note that these are cross-sectional associations, effect sizes are small-to-moderate, and our results could also be inflated due to shared method variance.

This study has several strengths. First, we examined both mental and physical health outcomes within the same sample of children, contributing to a more comprehensive understanding of the effects of nurturant-involved parenting on children’s health. Additionally, inclusion of the blood draw and physical measurements enabled us to capture objective clinical indicators of children’s physical health, representing an advance over previous studies that have relied on self-report health measures (Anderson et al., 2020). This study also addresses existing gaps in the literature by examining unique psychological stress in young children of color, advancing research on an understudied and historically marginalized population.

Although these findings represent an important contribution to our understanding of the role of context in shaping children’s mental and physical health, the present study also has several limitations, and it will be important for future research to replicate these findings. First, current analyses focus on cross-sectional evaluation of parenting, stress, discrimination, and mental and physical health. Future research should examine how these processes unfold over time to examine how changes in nurturant-involved parenting or stress exposures influence internalizing symptoms and cardiometabolic risk. Additionally, we relied solely on guardian reports of their nurturant-involved parenting behavior and children’s self-reports of stress, discrimination, and internalizing symptoms. Future research could benefit from utilizing a multi-method approach to assess these constructs, such as the addition of behavioral observations of nurturant-involved parenting. Another limitation of the present work is that the blood draws were nonfasting to accommodate participants’ schedules. Although we would have ideally asked participants to complete an overnight fast prior to the blood draw, evidence from adult samples suggests that nonfasting lipids do not vary dramatically after eating and still maintain clinical significance (Bansal et al., 2007; Darras et al., 2018). The majority (62%) of study visits occurred in the afternoon, which limits between-person variation in cardiometabolic markers that might be influenced by the time of day.

Although we had a wide age range within our sample, we were not powered to test whether the interactions between nurturant-involved parenting and stressors varied as a function of children’s age (though age was included as a control variable). Future research with larger sample sizes will be needed in order to examine how the benefits of nurturant-involved parenting for children’s health differ based on demographic variables such as age, race/ethnicity, and gender. For example, the types of stressful experiences that young children encounter may be significantly different from those that teens experience. Older children may therefore be more vulnerable to the negative effects of stress due to the increased severity of stressors in their daily lives. Conversely, it is possible that younger children may be more vulnerable to the effects of stress and discrimination because they have fewer internal resources and strategies for effective coping. Similarly, Black and Latinx children may face unique stressors and different forms of discrimination. For example, Latinx children may have worries related to language barriers or immigration, whereas Black children may be more concerned about stressors related to police brutality. Given the distinct experiences and challenges that each of these populations face, the forces driving these results may differ based on factors that are race- or ethnicity-specific.

## Conclusion

The health trajectories that emerge during childhood can compound and have lifelong consequences for individuals’ mental and physical well-being, underscoring the importance of understanding how parenting may influence health across the lifespan. The current study adds meaningful insights to existing literature about the role that parents can play in supporting their children’s health when youth are exposed to high levels of stress and discrimination. When parents engage in supportive behaviors, particularly when children are experiencing heightened levels of stress and discrimination, they may help promote children’s physical health. Our findings also highlight that the effects of parenting are context-dependent and underscore the importance of considering race and ethnicity-specific stressors such as discrimination when examining how children’s social worlds shape their health. Although parents can play an important role in promoting health when children are in distress, we must ultimately work to reduce the amount of stress and discrimination that children experience to promote children’s long-term psychological and physical well-being.

## Figures and Tables

**Table 1.** Descriptive Statistics and Correlations Among Study Variables

Variable	*M (SD)*	1	2	3	4	5	6	7	8	9
1. Age	11.52 (2.56)	—								
2. Sex	.49 (.50)	−.04	—							
3. Race	.41 (.50)	−.01	−.02	—						
4. SES Risk	2.57 (1.40)	−.03	−.09	−.22**	—					
5. Nurturant-Involved Parenting	3.33 (.46)	−.04	.07	.08	.04	—				
6. Stress	2.45 (.84)	−.17*	.07	.09	−.04	.08	—			
7. Discrimination	2.28 (1.08)	−.21**	−.07	−.10	.12	.02	.40**	—		
8. Internalizing Symptoms	.00 (.89)	−.12	.07	−.02	.03	.01	.61**	.45**	—	
9. Cardiometabolic Risk	1.02 (.93)	−.01	−.01	.02	.04	−.14^†^	−.05	−.05	−.09	—

*Note.* SES = socioeconomic status. Sex (0 = male, 1 = female); Race (0 = Black/African American, 1 = Hispanic/Latinx).

† *p* < .10

* *p* < .05

** *p* < .01.

**Table 2.** Regression analyses of nurturant-involved parenting and stress predicting internalizing symptoms and cardiometabolic risk in youth

	*b*	*SE*	95% *CI*
*Internalizing symptoms*			
Age	−0.01	0.02	[−.05, .04]
Sex	0.08	0.12	[−.15, .30]
Race	−0.13	0.12	[−.36, .11]
SES risk	0.03	0.04	[−.06, .11]
Nurturant-involved parenting	−0.09	0.13	[−.34, .15]
Stress	0.65**	0.07	[.52, .79]
Nurturant-involved parenting × stress	−0.13	0.09	[−.31, .05]
*Cardiometabolic risk*			
Age	−0.01	0.03	[−.07, .05]
Sex	0.06	0.15	[−.23, .38]
Race	0.08	0.15	[−.23, .38]
SES risk	0.03	0.05	[−.07, .14]
Nurturant-involved parenting	−0.32^†^	0.16	[−.64, .01]
Stress	−0.03	0.09	[−.20, .15]
Nurturant-involved parenting × stress	−0.37^†^	0.20	[−.76, .02]

*Note.* All analyses were conducted in SPSS using Hayes' PROCESS Macro Version 4.1. Race was dummy-coded as Black/African American (0)/Hispanic/Latinx (1). Sex was dummy-coded as male (0)/female (1).

† *p* < .10

** *p* < .01.

**Table 3.** Regression analyses of nurturant-involved parenting and discrimination predicting internalizing symptoms and cardiometabolic risk in youth

	*b*	*SE*	95% *CI*
*Internalizing symptoms*			
Age	−0.01	0.03	[−.06, .04]
Sex	0.17	0.13	[−.08, .42]
Race	0.06	0.13	[−.20, .32]
SES risk	−0.004	0.05	[−.10, .09]
Nurturant-involved parenting	−0.02	0.14	[−.30, .25]
Discrimination	0.38**	0.06	[.26, .50]
Nurturant-involved parenting × discrimination	−0.15	0.13	[−.42, .12]
*Cardiometabolic risk*			
Age	−0.01	0.03	[−.07, .05]
Sex	0.04	0.15	[−.25, .33]
Race	0.07	0.15	[−.24, .37]
SES risk	0.04	0.05	[−.07, .14]
Nurturant-involved parenting	−0.28^†^	0.16	[−.60, .03]
Discrimination	−0.05	0.07	[−.19, .09]
Nurturant-involved parenting × discrimination	−0.32*	0.16	[−.63, −.01]

*Note.* All analyses were conducted in SPSS using Hayes’ PROCESS Macro Version 4.1. Race was dummy-coded as Black/African American (0)/Hispanic/Latinx (1). Sex was dummy-coded as male (0)/female (1).

† *p* < .10

* *p* < .05

** *p* < .01.
